# Hot Water Remedies

**Published:** 1891-03

**Authors:** 


### Hot Water Remedies.

Headache almost always yields to the simultaneous application of hot water to the feet and back of the neck.

A towel folded, dipped in hot water, wrung out rapidly and applied to the stomach acts like magic in cases of colic.

There is nothing that so promptly cuts short congestion of the lungs, sore throat or rheumatism, as hot water when applied promptly and thoroughly.

A towel folded several times and dipped in hot water and wrung and applied over the toothache or neuralgia, will generally afford prompt relief.

A strip of flannel or napkin folded lengthwise and dipped in hot water and wrung out, and then applied to the neck of a child that has the croup, will usually bring relief in ten minutes.

Hot water taken freely half an hour before bed time is the best cathartic possible in the case of constipation, while it has a most soothing effect upon the stomach and bowels.—*Hall's Journal of Health.*

